# Factors That Influence Refractive Changes in the First Year of Myopia Development in Premature Infants

**DOI:** 10.1155/2019/7683749

**Published:** 2019-06-03

**Authors:** Jianbo Mao, Jimeng Lao, Chenyi Liu, Mingyuan Wu, Xueting Yu, Yirun Shao, Lin Zhu, Yiqi Chen, Lijun Shen

**Affiliations:** ^1^Eye Hospital of Wenzhou Medical University, Wenzhou, Zhejiang, China; ^2^Chicago College of Optometry, Midwestern University, Downers Grove, IL, USA; ^3^Women's Hospital School of Medicine Zhejiang University, Hangzhou, Zhejiang, China

## Abstract

**Purpose:**

To study the development of refractive status from 36 weeks to one year of postmenstrual age and to identify factors that contribute to development of myopia, including gender, birth weight, gestational age, and retinopathy of prematurity (ROP).

**Methods:**

Premature infants underwent full cycloplegic retinoscopy at 36 weeks, 38 weeks, 40 weeks, 42 weeks, 44 weeks, 46 weeks, 48 weeks, 3 months, 6 months, 9 months, and 12 months of postmenstrual age. The infants were grouped by gender, birth weight, gestational age, and the severity of ROP to evaluate the correlation with refractive status at each postmenstrual age.

**Results:**

A total of 942 infants were recruited in this study. A total of 2716 readings were obtained. Refractive state had a hyperopic shift until 46 weeks of postmenstrual age (*r* = 0.42, *P* < 0.0001). After that, the mean spherical equivalent (SE) gradually declined (*r* = −0.30, *P* < 0.0001). Boys had lower hyperopia than girls at nine months (*t* = 3.10, *P*=0.003) and one year (*t* = 3.34, *P*=0.001) of postmenstrual age. Premature infants with ROP had a lower average SE at most of the postmenstrual ages; however, this value did not vary significantly (*P* > 0.05). Premature infants with severe ROP were less hyperopic than those without it at every postmenstrual age, and the average SE differed significantly at one year of postmenstrual age (*t* = 2.60, *P*=0.011). There was no significant difference between each birth weight and gestational age (*P* > 0.05).

**Conclusions:**

The dioptric value of premature infants within one year was generally hyperopic. Different gender, birth weight, gestational age, and ROP did not affect the overall development of refractive status. Females may have higher hyperopia at nine months of postmenstrual age. Birth weight and gestational age had little effect on change of refractive status. Severe ROP was an important contributing factor in myopia progression, which may be related to the treatment required. Further study may be carried out to understand the mechanism behind myopia progression in premature infants, including changes in refractive system parameters and emmetropization process.

## 1. Introduction

With the improvement of neonatal care technology, the number of premature infants is increasing. Prematurely born children are at increased risk of visual disability. A number of studies have reported presence of refractive errors to be more common in prematurely born children [1–4]. It has been suggested that gender, birth weight, gestational age, and retinopathy of prematurity (ROP) may be associated with myopia [5–7]. However, these studies mainly focused on the first year of life. There is a lack of large-sample studies on the early postnatal period.

It remains controversial whether either ROP disease or ROP treatment or both of them are myopia-related factors. The two correlative factors have been analyzed, however, without control groups at the same postmenstrual age. This may lead to inexact results. Studies that analyzed large-sample of patients at the same postmenstrual age are still rare.

Our purpose was to study the development of refractive status from 36 weeks to one year of postmenstrual age and to identify factors that contribute to the development of myopia, including gender, birth weight, gestational age, and ROP.

## 2. Materials and Methods

A total of 942 premature infants who were screened at the hospital for retinopathy of prematurity (ROP) participated in this study. The exam was performed at the Eye Hospital of Wenzhou Medical University from January 2016 to May 2018. Regional Ethics Committee approval and parental consent were obtained in each case. Infants with poor physical condition leading to intolerance of examination, infectious disease, and refractive interstitial opacity such as congenital cataracts were excluded from this study.

The eye examinations were performed respectively at 36 weeks, 38 weeks, 40 weeks, 42 weeks, 44 weeks, 46 weeks, 48 weeks, 3 months, 6 months, 9 months, and 12 months of postmenstrual age. All methodologies adhered to the tenets of the Declaration of Helsinki.

One drop of 5 mg/ml topical tropicamide was instilled three times in each eye at an interval of 10 minutes. Full cycloplegic retinoscopy was performed 30 minutes after the last instillation using a streak retinoscope (66 Vision Technology, Suzhou, Jiangsu Province, China). Eyelids were held open with a pediatric speculum after topical anesthesia with 1 drop of 0.5% proparacaine hydrochloride. Retinoscopy was performed by the same examiner (first author in this study, senior optometrist) at the Eye Hospital of Wenzhou Medical University, Hangzhou, China. An allowance of 2.00 diopters (D) was made for a working distance of 50 centimeters. Refractive error was recorded in the form of spherical equivalent (SE), where the SE = sphere + cylinder/2. The basic information, including gender, birth weight, gestational age, postmenstrual age, and presence of ROP, was recorded at each follow-up. Premature infants were classified by gender, birth weight, gestational age, and severity of ROP to evaluate the correlation between these parameters and refractive status. ROP that required treatment was defined as severe ROP.

The right eye of each infant was used for further data collection and analysis. Statistical analysis was performed using the Statistical Package for Social Sciences (SPSS version 19.0, SPSS, Inc., Chicago, IL, USA). The results were recorded as the means ± standard deviations (SD). Correlation analysis was used to analyze the relationship between SE and postmenstrual age. Independent samples *t*-tests were used to evaluate the correlation between these parameters (gender, birth weight, gestational age, and presence or absence ROP or severe ROP) and refractive status at each postmenstrual age. Significance was defined as *P* < 0.05.

## 3. Results

A total of 942 infants (427 females, 515 males) were recruited in this study. The average gestational age was 30.3 ± 2.3 weeks (range, 26–36 weeks). The average birth weight was 1,485.8 ± 401.8 g (range, 740–3,390 g). A total of 2716 readings were obtained.

**Figure 1:**
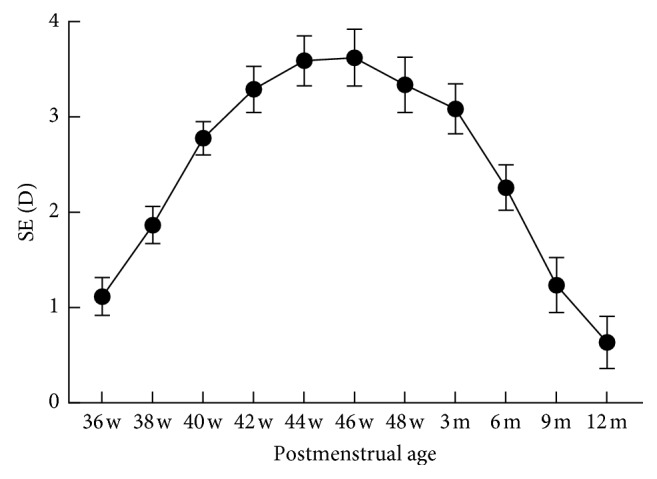
Correlation between SE and postmenstrual age. SE, spherical equivalent; D, diopter.

The mean SE was +(1.12 ± 2.10) D at 36 weeks of postmenstrual age followed by a hyperopic shift until the SE reached +(3.62 ± 1.77) D at 46 weeks of postmenstrual age (*r* = 0.42, *P* < 0.0001). After that, the mean SE gradually declined and reached +(0.64 ± 1.12) D at one year of postmenstrual age (*r* = −0.30, *P* < 0.0001) (Table 1; Figure 1).

**Figure 2:**
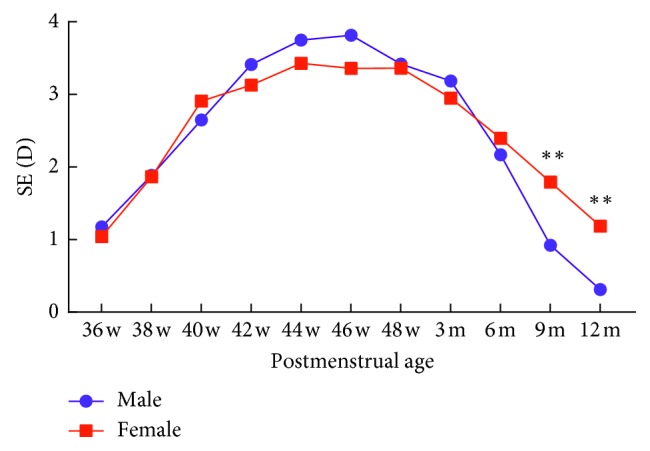
Correlation between SE and gender. SE, spherical equivalent; D, diopter.

The mean SE of boys was more hyperopic than that of girls during the early period. Beginning from 6 months of postmenstrual age, the SE of boys rapidly decreased with a myopic shift (Table 2; Figure 2). There was a significant difference at nine months (*t* = 3.10, *P*=0.003) and one year (*t* = 3.34, *P*=0.001) of postmenstrual age.

**Figure 3:**
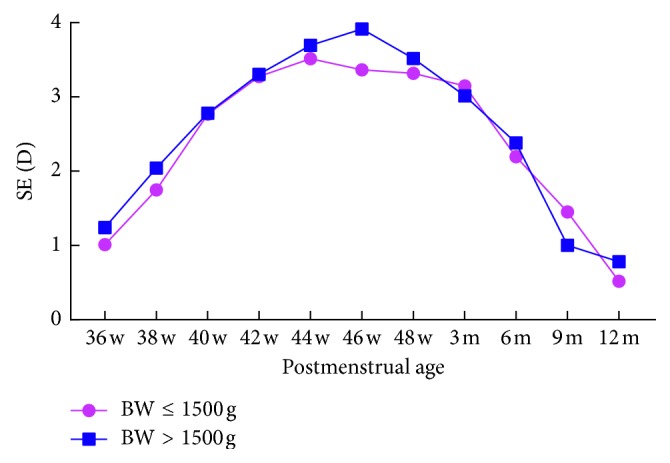
Correlation between SE and BW. SE, spherical equivalent; D, diopter; BW, birth weight.

**Figure 4:**
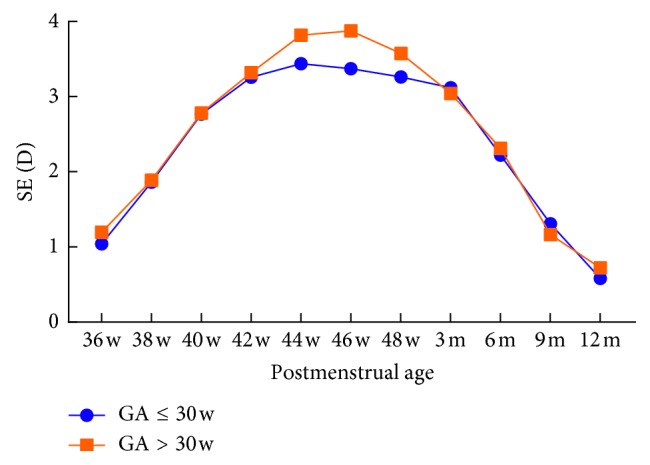
Correlation between SE and GA. SE, spherical equivalent; D, diopter; GA, gestational age.

Infants were grouped according to gestational age (GA **≤** 30 weeks; GA > 30 weeks) and birth weight (BW **≤** 1500 g; BW > 1500 g) to evaluate the correlation between refractive status and the two variables, respectively. At the same postmenstrual age, there was no significant difference between the groups (*P* > 0.05) (Tables 3 and 4; Figures 3 and 4).

**Figure 5:**
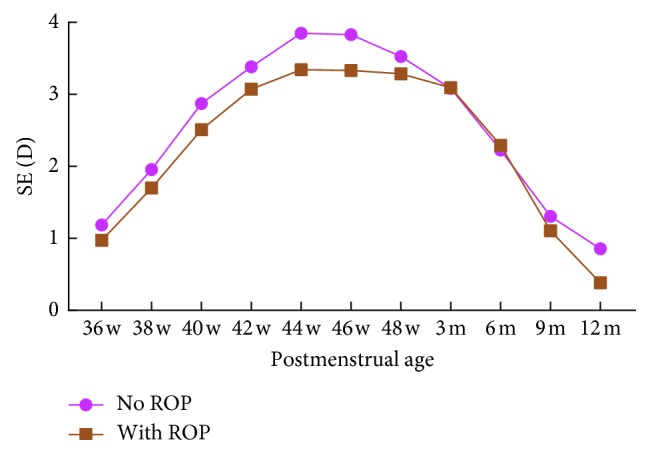
Correlation between SE and ROP. SE, spherical equivalent; D, diopter.

**Figure 6:**
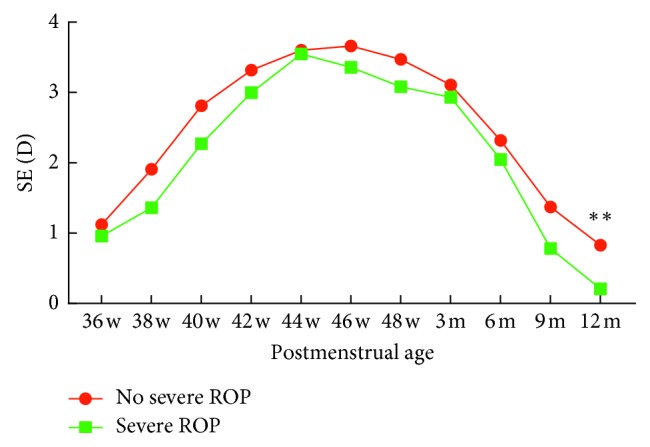
Correlation between SE and severe ROP. SE, spherical equivalent; D, diopter.

The average SEs at each postmenstrual age with and without presence of ROP are presented in Table 5 and Figure 5. Premature infants with ROP were more myopic than those without ROP at most of the postmenstrual ages; however, the difference was not significant (*P* > 0.05). Premature infants with severe ROP were more myopic than those without severe ROP at each postmenstrual age. The average SE differed significantly at one year of postmenstrual age (*t* = 2.60, *P*=0.011) (Table 6; Figure 6).

## 4. Discussion

In our research, the mean refractive index was +1.12 D at 36 weeks of postmenstrual age followed by a hyperopic shift until the refractive index reached +3.62 D at 46 weeks of postmenstrual age. Then, the mean SE gradually declined and reached +0.64 D at one year of postmenstrual age. In the study by Yu et al. [8], the refractive index reached the maximum value (+2.43 ± 1.46 D) at the age of 1-2 months and then declined. At the age of one year, the refractive index reached +0.59 D. Although full-term infants were also included in research, their results were similar to ours, which suggested that there might be little difference in refractive errors between full-term infants and preterm infants at the same postmenstrual age. In this study, the earliest examination interval of premature infants was at two weeks after birth. The SE reached maximum value at 46 weeks of postmenstrual age. The peak period was also consistent in gender, BW and GA groups. This is of great significance for the future study on the development of refractive index in premature infants and full-term infants. The correlation between refractive error and gender is still controversial [8–12]. Yu et al. [8] found that boys had higher hyperopia than girls during the first year of life, while Ozdemir et al. [12] found that both the spherical equivalent and astigmatic error were similar in female and male infants. In the present paper, we found that females had higher hyperopia than males at nine months and one year of postmenstrual age, which suggested that males had a greater susceptibility to develop myopia. It is also possible that the emmetropization was reached earlier in boys than girls. Longer follow-up period will be required to evaluate the development of emmetropization in premature infants.

Premature infants were reported to be more susceptible to myopia development. Through a 10-year follow-up, Larsson and Holmstrom [13] found that the risk of vision impairment and significant refractive error in preterm infants was significantly higher than in full-term infants. Abnormal development of the refractive system may explain the myopia progression in prematurity. Studies [13, 14] have found that premature infants with myopia have greater corneal curvature, shallower anterior chamber depth, thicker lens, and shorter axial length compared to normal full-term infants. Further study is required to fully understand the specific mechanism.

Studies [5–7] on the influencing factors of refractive status early in life have found that preterm babies with lower birth weight and lower gestational age had lower spherical equivalent. Varughese et al. [15] pointed out in their study that preterm infants with a lower gestational age can be either hyperopic or myopic, while preterm infants with a higher gestational age tended to be more hyperopic. Statistics showed that the refractive state of preterm infants was related to their gestational age, birth weight, head size, and body length. We believe that gestational age at birth only casts an early influence on the dioptric value of preterm infants. Influencing factors during later period need to be explored at different postmenstrual ages, given that the preterm infants are typically at various developmental stages. Modrzejewska et al. [16] found that, at 64 weeks of postmenstrual age, infants with birth weights ranging from 1,556 g to 1,621 g were hyperopic, while those with birth weights from 810 g to 1,234 g were myopic. Some long-term studies [17, 18] have shown that low birth weight is a risk factor for myopia development. The risk of myopia at 12 months of age showed a threefold increase with a birth weight less than 751 g [19]. In contrast, Ton et al. [20] reported that gestational age and birth weight had no impact on the refractive status in the preterm babies. In this research, although babies with a birth weight of more than 1500 g had a greater mean SE for most postmenstrual ages, there was no significant difference in refractive error between those with a birth weight of less than 1500 g and those with a birth weight of more than 1500 g. Differences with the previous studies may be related to the threshold of birth weight used for grouping. For the gestational age, the previous results were relatively consistent. It is believed that the gestational age had little effect on the refractive error.

ROP is an important factor leading to myopia progression. Previous research [21, 22] found that severe ROP or ROP requiring treatment can cause high myopia, while mild ROP or spontaneously regressed ROP did not cause high myopia. Kaya et al. [21] studied 224 premature infants who were followed up to age 6 and found that premature infants with severe ROP were more likely to develop myopia and astigmatism. Kuo et al. [22] studied 54 eyes and found that the mean spherical equivalent at 3 years of age was –1.71 ± 1.27 D in patients treated with laser therapy, –1.53 ± 2.20 D in patients treated with intravitreal injection of bevacizumab, +0.63 ± 1.37 D in patients with ROP that does not require treatment, and +0.41 ± 1.95 D in premature infants without ROP. Overall, patients needing treatment were more prone to myopia compared to those without treatment. Some scholars [23] have found that children with ROP after treatment have a higher risk of developing astigmatism at 3 years after birth, mainly due to retinal scar formation after laser treatment. Quinn et al. [24] believed that the development of myopia was also related to the formation of scar in the lesion. Other scholars [25], however, stated that it was the severity of ROP that resulted in the high incidence of myopia rather than the treatment that was required for the severeness of the disease. In our research, premature infants with ROP were more myopic than those without ROP at most of the postmenstrual ages; however, this value did not vary significantly. Additionally, premature infants with severe ROP were more myopic than those without severe ROP at each postmenstrual age. The average SE differed significantly at one year of postmenstrual age. The results suggested that ROP disease has little effect on refractive status, and ROP treatment is a risk factor for myopia development. Statistical differences that did not appear until one year after birth were most likely directly related to treatment.

## 5. Conclusions

The refractive state had a hyperopic shift until 46 weeks of postmenstrual age. After that, the mean dioptric value gradually and continuously declined by one year of postmenstrual age. In general, the dioptric value of premature infants within one year is generally hyperopic. Different gender, birth weight, gestational age, and ROP did not affect the overall development of refractive status. Gender may not have an effect on dioptric values at an early age; however, females may have higher hyperopia at nine months of postmenstrual age. Birth weight and gestational age had little effect on change of refractive status. Severe ROP is an important contributing factor in myopia progression, which may be related to the treatment required.

The relationship between dioptric value and refractive system parameters, such as axial lengths and corneal curvature, is expected to be further studied to aid in understanding the mechanism of myopia development. We will also conduct a long-term follow-up study on premature infants to observe the emmetropization process, to discuss the physiological reference value for development of refractive state in Chinese premature infants at different stages and to understand the time point for emmetropization, all of which will be valuable in preventing myopia progression.

## Figures and Tables

**Table 1:** SEs from 36 weeks to one year of postmenstrual age.

Postmenstrual age	*n*	Refraction (D)
36 w	437	+1.12 ± 2.10
38 w	411	+1.88 ± 1.89
40 w	382	+2.78 ± 1.73
42 w	213	+3.29 ± 1.78
44 w	144	+3.59 ± 1.59
46 w	137	+3.62 ± 1.77
48 w	102	+3.40 ± 1.46
3 m	146	+3.09 ± 1.60
6 m	199	+2.26 ± 1.71
9 m	69	+1.24 ± 1.20
12 m	67	+0.64 ± 1.12

SE, spherical equivalent; *w*, weeks; *m*, months; *n*, number of eyes; D, diopter.

**Table 2:** SE of different genders.

Postmenstrual age	Males		Females		*t*	*P*
	*n*	SE (D)	*n*	SE (D)		
36 w	243	+1.18 ± 2.12	194	+1.04 ± 2.08	0.67	0.505
38 w	234	+1.88 ± 1.94	177	+1.87 ± 1.82	0.10	0.920
40 w	193	+2.65 ± 1.73	189	+2.91 ± 1.73	1.46	0.145
42 w	121	+3.41 ± 1.97	92	+3.13 ± 1.48	1.16	0.246
44 w	75	+3.75 ± 1.56	69	+3.43 ± 1.62	1.21	0.229
46 w	79	+3.82 ± 1.74	58	+3.36 ± 1.77	1.51	0.133
48 w	62	+3.42 ± 1.48	40	+3.36 ± 1.41	0.18	0.855
3 m	85	+3.19 ± 1.60	61	+2.95 ± 1.59	0.89	0.376
6 m	119	+2.17 ± 1.58	80	+2.39 ± 1.87	0.92	0.359
9 m	44	+0.92 ± 0.99	25	+1.79 ± 1.33	3.10	0.003
12 m	42	+0.31 ± 0.96	25	+1.19 ± 1.16	3.34	0.001

SE, spherical equivalent; *w*, weeks; *m*, months; *n*, number of eyes; D, diopter.

**Table 3:** SE of different BW.

Postmenstrual age	BW ≤ 1500 g		BW > 1500 g		*t*	*P*
	*n*	SE (D)	*n*	SE (D)		
36 w	240	+1.18 ± 2.12	197	+1.24 ± 2.19	1.14	0.257
38 w	231	+1.75 ± 1.89	180	+2.04 ± 1.88	1.57	0.117
40 w	172	+2.65 ± 1.73	210	+2.78 ± 1.72	0.08	0.937
42 w	108	+3.41 ± 1.97	105	+3.31 ± 1.82	0.12	0.902
44 w	81	+3.75 ± 1.56	63	+3.70 ± 1.81	0.68	0.500
46 w	73	+3.82 ± 1.74	64	+3.91 ± 1.84	1.84	0.069
48 w	63	+3.32 ± 1.42	39	+3.52 ± 1.50	0.55	0.676
3 m	78	+3.19 ± 1.60	68	+3.02 ± 1.65	0.50	0.620
6 m	119	+2.17 ± 1.58	80	+2.38 ± 1.81	0.76	0.445
9 m	36	+0.92 ± 0.99	33	+1.00 ± 1.20	1.60	0.116
12 m	37	+0.31 ± 0.96	30	+0.78 ± 0.98	0.97	0.335

BW, birth weight; SE, spherical equivalent; *w*, weeks; *m*, months; *n*, number of eyes; D, diopter.

**Table 4:** SE of different GA.

Postmenstrual age	GA ≤ 30 w		GA > 30 w		*t*	*P*
	*n*	SE (D)	*n*	SE (D)		
36 w	226	+1.04 ± 2.00	211	+1.20 ± 2.21	0.76	0.447
38 w	204	+1.86 ± 1.84	207	+1.89 ± 1.93	0.14	0.885
40 w	159	+2.77 ± 1.66	223	+2.78 ± 1.79	0.08	0.938
42 w	101	+3.26 ± 1.60	112	+3.32 ± 1.92	0.23	0.815
44 w	85	+3.44 ± 1.41	59	+3.82 ± 1.81	1.43	0.156
46 w	69	+3.37 ± 1.75	68	+3.88 ± 1.75	1.68	0.095
48 w	58	+3.26 ± 1.41	44	+3.57 ± 1.50	1.08	0.282
3 m	84	+3.12 ± 1.58	62	+3.04 ± 1.63	0.29	0.769
6 m	118	+2.22 ± 1.64	81	+2.31 ± 1.80	0.37	0.715
9 m	34	+1.31 ± 1.18	35	+1.16 ± 1.21	0.50	0.616
12 m	41	+0.58 ± 1.18	26	+0.72 ± 1.01	0.50	0.622

GA, gestational ages; SE, spherical equivalent; *w*, weeks; *m*, months; *n*, number of eyes; D, diopter.

**Table 5:** SE of the presence or absence of ROP.

Postmenstrual age	No ROP		With ROP		*t*	*P*
	*n*	SE (D)	*n*	SE (D)		
36 w	296	+1.18 ± 2.21	141	+0.97 ± 1.84	0.98	0.327
38 w	284	+1.96 ± 1.96	127	+1.70 ± 1.72	1.29	0.199
40 w	283	+2.87 ± 1.75	99	+2.51 ± 1.65	1.78	0.075
42 w	150	+3.38 ± 1.86	63	+3.07 ± 1.56	1.17	0.244
44 w	72	+3.85 ± 1.69	72	+3.34 ± 1.45	1.93	0.056
46 w	80	+3.83 ± 1.78	57	+3.33 ± 1.71	1.63	0.105
48 w	47	+3.52 ± 1.38	55	+3.28 ± 1.51	0.83	0.411
3 m	80	+3.08 ± 1.52	66	+3.09 ± 1.70	0.04	0.970
6 m	97	+2.22 ± 1.71	102	+2.29 ± 1.70	0.28	0.779
9 m	45	+1.31 ± 1.16	24	+1.10 ± 1.26	0.67	0.505
12 m	36	+0.85 ± 1.04	31	+0.38 ± 1.16	1.75	0.084

ROP, retinopathy of prematurity; SE, spherical equivalent; *w*, weeks; *m*, months; *n*, number of eyes; D, diopter.

**Table 6:** SE of the presence or absence of serious ROP.

Postmenstrual age	No severe ROP		Severe ROP		*t*	*P*
	*n*	SE (D)	*n*	SE (D)		
36 w	425	+1.12 ± 2.12	12	+0.96 ± 1.59	0.26	0.795
38 w	383	+1.91 ± 1.88	28	+1.36 ± 1.91	1.49	0.137
40 w	359	+2.81 ± 1.74	23	+2.27 ± 1.54	1.45	0.148
42 w	195	+3.32 ± 1.81	18	+3.00 ± 1.38	0.73	0.466
44 w	118	+3.60 ± 1.64	26	+3.55 ± 1.35	0.14	0.885
46 w	121	+3.66 ± 1.81	16	+3.36 ± 1.41	0.64	0.524
48 w	83	+3.47 ± 1.46	19	+3.08 ± 1.40	1.06	0.292
3 m	127	+3.11 ± 1.53	19	+2.93 ± 2.02	0.46	0.648
6 m	155	+2.32 ± 1.71	44	+2.05 ± 1.67	0.93	0.353
9 m	53	+1.37 ± 1.23	16	+0.78 ± 0.95	1.76	0.082
12 m	46	+0.83 ± 1.04	21	+0.21 ± 1.17	2.17	0.033

ROP, retinopathy of prematurity; SE, spherical equivalent; *w*, weeks; *m*, months; *n*, number of eyes; D, diopter.

## Data Availability

The data used to support the findings of this study are available from the corresponding author upon request.
